# The Rage for Invention

**Published:** 1843-06

**Authors:** 


*The Rage for Invention.*—Velpeau and Blandin have both deprecated the present mania for the invention of new surgical instruments in France, as if surgery were not already overladen with mechanical contrivance. Velpeau attributes this mania, which prevails mostly among young surgeons, to the ignorance of these youths as to what has been done before them; and he judiciously observes, that "it would be much better for them to study the capacities of the surgical instruments that at present exist than to invent others of which there is no need."—*London Lancet.*

